# The influence of invasive growth pattern and microvessel density on prognosis in colorectal cancer and colorectal liver metastases

**DOI:** 10.1038/sj.bjc.6603677

**Published:** 2007-03-13

**Authors:** R Rajaganeshan, R Prasad, P J Guillou, C R Chalmers, N Scott, R Sarkar, G Poston, D G Jayne

**Affiliations:** 1 Academic Surgical Unit, St James's University Hospital, Leeds, LS9 7TF, UK; 2 Department of Hepatobiliary and Transplant Surgery, St James's University Hospital, Leeds, LS9 7TF, UK; 3 Department of Pathology, St James's University Hospital, Leeds, LS9 7TF, UK; 4 Liverpool Hepato-Biliary Centre, University Hospital Aintree, Liverpool, L9 7AL, UK

**Keywords:** microvessel density, colorectal cancer, liver metastases, invasion

## Abstract

The nature of the invasive growth pattern and microvessel density (MVD) have been suggested to be predictors of prognosis in primary colorectal cancer (CRC) and colorectal liver metastases. The purpose of the present study was to determine whether these two histological features were interrelated and to assess their relative influence on disease recurrence and survival following surgical resection. Archival tissue was retrieved from 55 patients who had undergone surgical resection for primary CRC and matching liver metastases. The nature of the invasive margin was determined by haematoxylin and eosin (H&E) histochemistry. Microvessel density was visualised using immunohistochemical detection of CD31 antigen and quantified using image capture computer software. Clinical details and outcome data were retrieved by case note review and collated with invasive margin and MVD data in a statistical database. Primary CRCs with a pushing margin tended to form capsulated liver metastases (*P*<0.001) and had a significantly better disease-free survival than the infiltrative margin tumours (log rank *P*=0.01). Primary cancers with a high MVD tended to form high MVD liver metastases (*P*=0.007). Microvessel density was a significant predictor of disease recurrence in primary CRCs (*P*=0.006), but not liver metastases. These results suggest that primary CRCs and their liver metastases show common histological features. This may reflect common mechanisms underlying the tumour–host interaction.

Approximately 50% of patients with colorectal cancer (CRC) will be cured by surgical resection. The remainder will develop disease recurrence, with the liver being the commonest site for metastatic spread (Poston, 2004). In selected patients, liver metastases may be amenable to surgical resection with 5-year survival rates of 35–40% (Lodge *et al*, 2005). For those with unresectable liver metastases, or who recur after liver resection, the prognosis is poor.

Several histological variables have been identified as predictors of disease recurrence, including the degree of tumour differentiation, the depth of local invasion, the presence of nodal metastases, and evidence of extramural vascular or perineural invasion. In 1987, Jass *et al* (1987) identified the nature of the invasive growth pattern as a prognostic indicator in CRC, with infiltrative cancers having a worse prognosis compared to pushing cancers. The invasive growth pattern of colorectal liver metastases was subsequently shown to predict outcome following liver resection, with capsulated and noncapsulated metastases being associated with 5-year survival rates of 76 and 31%, respectively (Okano *et al*, 2000). However, it is not known whether the invasive growth patterns of the primary cancers and their liver metastases are related, or whether the growth pattern is a genetically determined feature of the tumour, a response to local tumour–stromal interaction, or a combination of the two.

Angiogenesis is a fundamental requirement for tumour growth, invasion, and metastasis formation (Folkman, 1986). The assessment of microvessel density (MVD) is frequently used to quantify angiogenesis in archival tissue (Hlatky *et al*, 2002), although its use as a prognostic indictor is controversial. In breast cancer (Uzzan *et al*, 2004), non-small cell lung carcinoma (Ushijima *et al*, 2001) and melanoma (Ribatti *et al*, 2003), a high MVD is associated with poor outcome. In CRC, a high MVD has been shown to predict disease recurrence and survival in some studies (Saclarides *et al*, 1994;Tanigawa *et al*, 1997), whereas other studies have shown an association between high MVD and improved survival (Prall *et al*, 2003) or have concluded that MVD failed to provide additional prognostic information (Cianchi *et al*, 2002).

The nature of the invasive growth pattern and the angiogenic response both influence a tumour's metastatic capability. However, it is not known if these processes are interrelated. It is possible that different invasive phenotypes are associated with different angiogenic responses, which in turn influence their metastatic capability. The current study aimed to investigate the relationship between the invasive growth patterns observed in primary CRC and liver metastases, the association with MVD, and the impact on clinical outcome.

## MATERIALS AND METHODS

Patients who had undergone potentially curative resection for CRC and colorectal liver metastases were identified from histopathology databases at St James's University Hospital, Leeds and the Royal Liverpool University Hospital, Liverpool, between June 1994 and June 2000. Patients with conditions known to predispose to CRC (e.g. inflammatory bowel disease, familial adenomatous polyposis), and those who had undergone preoperative chemo-radiotherapy were excluded. Patients who died as a complication of their surgery were excluded as their death was unrelated to tumour biology and introduced a confounding influence on survival analysis. Patients included had primary CRC resected and were either diagnosed with synchronous liver metastases and underwent resection or developed metachronous liver disease on follow-up surveillance and underwent resection. A large proportion of patients in the study had synchronous metastases. Demographics are shown in Table 1 for the total 55 patients included.

Local ethics committee approval was obtained for the study (Leeds West Research Ethics Committee, RL04/6669). Patient demographics were entered into a statistical database (SPSSv12.0, Chicago, IL, USA) and collated with clinical and histological data.

### Histochemical analysis of the invasive margin

Archival paraffin-embedded tissue samples of matched primary CRC and liver metastases were retrieved for all patients. Thin sections of 4 *μ*m were prepared, dewaxed in xylene, rehydrated in graded ethanol solutions, and stained with haematoxylin and eosin (H&E) using a standard protocol.

The nature of the invasive margin was determined by two- independent observers and checked by a consultant histopathologist (NS). The invasive margin of the primary cancers were classified as either pushing or infiltrative based on the predominant morphology, as defined by Jass *et al* (1987). A modified classification based on Yasui (Lunevicius *et al*, 2001) was used for liver metastases, with metastases being classified *as* capsulated if >50% of the margin exhibited a fibrous capsule separating tumour from stroma.

### MVD analysis

Sections of 4 *μ*m sections were dewaxed in Histoclear (National Diagnostics, UK), rehydrated through graded ethanol, and washed with tap water for 10 min. Endogenous peroxidase activity was blocked with 0.6% (v v^−1^) H_2_O_2_ in 100% methanol for 10 min at room temperature. Antigen was retrieved by pressure cooking for 2 min in citrate buffer solution (pH 6.0). Sections were blocked with 5% (v v^−1^) goat serum (Sigma Aldrich, Dorset, UK) in phosphate-buffered saline (PBS) for 30 min at room temperature, and then incubated with anti-CD31 antibody (Dakocytomation, Ely, Cambridgeshire, UK) at 1 : 20 dilution in PBS overnight at 4°C. Sections were washed twice with PBS and then incubated with alkaline phosphatase–conjugated goat anti-mouse IgG (1 : 200 dilution; Dakocytomation, Ely, UK) for 60 min at room temperature. Following two further washes in PBS, the substrate was developed using the Vector Red ALP kit (Vector Laboratories, Peterborough, UK). Sections were counterstained with methyl green (Vector Laboratories, Peterborough, UK) for 10 min, washed in acetone, and dehydrated in a series of graded ethanol and Histoclear. Slides were mounted in diphenylxylene (BDH, Poole, UK) and cover slipped. Negative controls were performed in parallel, with omission of the primary antibody incubation step.

Anti-CD31 treated sections were examined with a Nikon Eclipse, (Kingston-upon-Thames, UK), E1000 microscope. Each section was divided at low power (× 40 optical magnification) into three areas representing the stromal interface, tumour margin, and the tumour centre. Five random high-power fields (× 100 optical magnification) were captured from each of the three tumour areas using a Basler A113™ Matrix-camera (Basler Vision Technology, UK) connected to a computer running Lucia G™ software (Version 4.6 Nikon). A template was created by setting a colour threshold, which clearly identified the CD31-positive endothelial cells and a fixed light intensity was used throughout the analysis. MVD counts were recorded from each of the five high-power fields and averaged to give the mean MVD at the stromal interface, the tumour margin, and the tumour centre. The MVD was calculated as the number of vessels per mm^2^. Twenty tissue samples were reanalysed 2 months following the initial analysis to assess intra and inter observer variation.

### Statistical analysis

Statistical analysis was performed using SPSS for windows (SPSS v12.0, CA, USA). The relationship between the invasive growth patterns of the primary CRCs and their liver metastases was tested using the *χ*^2^ test. The association between MVD and invasive growth pattern was analysed using the Mann–Whitney *U*-test. Spearman's correlation was performed to investigate the relationship of MVD in the primary cancer with that in the liver metastases and inter/intra observer variation. The difference in mean MVD between cancers that formed liver metastases and those that did not was tested using the Mann–Whitney *U*-test. The influence of mean MVD on disease-free and overall survival was analysed using Kaplan–Meier survival curves and log rank test. For all analyses a *P* value of <0.05 was deemed to be significant.

## RESULTS

Routine histological analyses of the resected primary CRCs are shown in Table 1. Twenty-nine (53%) patients were found to have synchronous liver metastases, whereas 26 (47%) patients developed metachronous liver disease. None of the patients had synchronous primary CRCs.

The mean length of follow-up for all patients was 47 months (range 6–171 months). A total of 28/55 (51%) patients were alive on completion of the study. The mean overall survival following resection of the primary cancers was 47 months (range: 6–171 months) and 36.3 months (range: 0–118 months) following liver resection. The mean disease-free survival following resection of primary cancers was 30 months (3–88 months) and 28 months (4–118 months) following liver resection. A total of 17/55 (30.9%) patients developed disease recurrence following liver resection: recurrence at the primary site (2), liver recurrence (7), lung recurrence (5), and multisite recurrence (3).

The scoring system for MVD was shown to be reliable and reproducible with significant correlations between the inter-observer (*r*=0.75, *P*<0.001) and intra-observer (*r*=0.71, *P*<0.01) variation when 20 sections were re-scored.

### Relationship between the invasive growth patterns in matched primary CRCs and liver metastases

**Figure 1:**
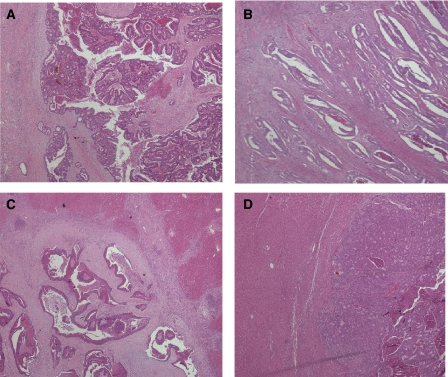
(**A**) Photomicrograph of a typical ‘pushing margin’ primary CRC. Original magnification was × 20. The H&E shows the penetration of normal tissues as a single advancing front. (**B**) Photomicrograph of a typical ‘infiltrative margin’ primary CRC. Original magnification was × 20. The H&E shows the diffuse manner of invasion in this margin, with widespread penetration of normal tissues. (**C**) Photomicrograph of a typical ‘capsulated’ colorectal liver metastases. Original magnification was × 20. The H&E shows a fibrous capsule separating the tumour from stroma. (**D**) Photomicrograph of a typical ‘noncapsulated’ colorectal liver metastases. Original magnification was × 20. The H&E shows the adjacent tumour and stroma with no fibrous capsule.

Twenty-six (47%) of the primary cancers had a pushing and 29 (53%) an infiltrative growth pattern (Figure 1A and B). A total of 18/26 (69.2%) primary cancers with a pushing growth pattern formed liver metastases with a capsulated phenotype (Figure 1C) compared to only 5/29 (17.2%) primary cancers with an infiltrative growth pattern (*χ*^2^, *P*<0.001). Similarly, primary cancers with an infiltrative growth pattern were significantly more likely to form noncapsulated metastases (Figure 1D) (*P*<0.001).

Colorectal cancers with a pushing margin had a significantly better disease-free survival than the infiltrative margin tumours (log rank *P*=0.017). Significant differences were not observed in overall survival (log rank *P*=0.10). Similarly capsulated and noncapsulated liver metastases in terms of disease-free survival (log rank *P*=0.32) or overall survival (log rank *P*=0.26).

### Relationship between MVD in matched primary CRC and liver metastases

**Figure 2:**
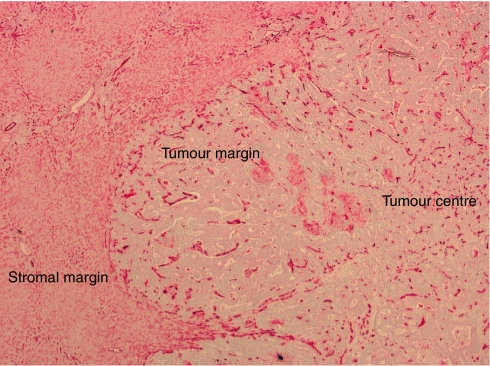
Colorectal cancer liver metastases stained with anti-CD31 antibody. The mean MVD was calculated at the stromal margin, tumour margin, and tumour centre.

**Figure 3:**
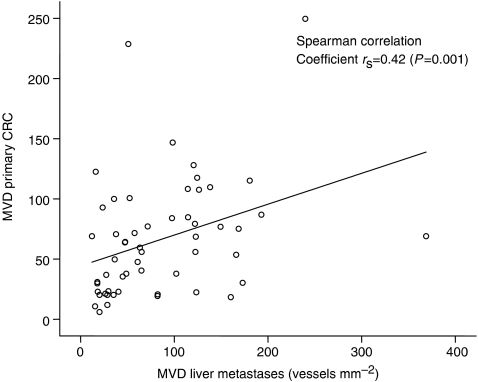
Correlation between MVD at the tumour margin of matched primary CRCs and liver metastases (vessels mm^−2^).

The mean MVD was determined at the stromal interface, the tumour margin, and the tumour centre for each CRC and liver metastasis (Figure 2). Primary cancers with a high MVD at the tumour margin tended to form liver metastases with a high MVD margin, whereas primary cancers with a low margin MVD tended to form metastases with a low MVD margin (*P*=0.001) (Figure 3). A similar positive correlation was observed when the primary and liver tumour centres were compared (*P*=0.011), but not on comparison of the stromal margins (*P*=0.149).

**Figure 4:**
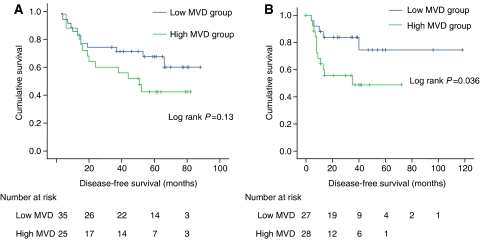
(**A**) Kaplan–Meier curve for disease-free survival following resection of primary CRC *vs* MVD expression divided into high and low MVD. (**B**) Kaplan–Meier curve for disease-free survival following resection of colorectal liver metastases *vs* MVD expression divided into high and low MVD.

The cohort was divided into high and low groups using the mean MVD values. The primary CRCs had a mean MVD of 65.2 vessels mm^−2^ (95% CI: 52.3–79.5), whereas the liver metastases had a mean MVD of 84.9 vessels mm^−2^ (95% CI: 66.8–103.1) at the tumour margin. The disease-free survival for the high and low MVD groups were not found to be significant following resection of primary CRC (log rank *P*=0.13) (Figure 4A), but significant following resection of colorectal liver metastases (log rank *P*=0.036) (Figure 4B).

### Relationship between invasive growth pattern and MVD in primary CRCs and liver metastases

When mean MVD measurements taken from the stromal interface, the tumour margin, and the tumour centre were compared between pushing and infiltrative primary cancers, a significant difference was only observed in the margin MVD (Table 2). Pushing cancers were associated with a higher margin MVD (mean: 82.4 vessels mm^−2^, 95% CI: 58.6–106.2) compared to infiltrative cancers (mean: 51.3 vessels mm^−2^, 95% CI: 38.9–63.7*, P*=0.034). A similar, but nonsignificant, trend to higher MVD was observed in the centre of pushing compared to infiltrative cancers (*P*=0.077, Table 2). Differences in MVD between capsulated and noncapsulated liver metastases were not significant at the stromal interface, tumour margin, or tumour centre.

## DISCUSSION

The prognosis and choice of therapy for patients with CRC is based on histological analysis and tumour stage. Jass *et al* (1987) identified the invasive margin in primary CRCs as a prognostic indicator, and similarly Okano *et al* (2000) showed that the growth pattern of colorectal liver metastases also predicted outcome. We have shown that pushing CRCs tend to develop capsulated liver metastases, whereas infiltrative cancers tended to develop noncapsulated liver metastases. Better disease-free survival was seen in primary cancers with a pushing margin, and this concurs with the previously reported finding of Jass *et al* (1987) and Cianchi *et al* (1997). However, unlike Okano no survival benefit was demonstrated between patients with capsulated and noncapsulated liver metastases. To date, no other group has compared the primary cancer phenotype with the invasive growth pattern of subsequent liver metastases.

Only the tumour margin MVD was found to be significant during the analysis of colorectal primary and liver metastases. Therefore, only the margin MVD will be used in further discussion.

Tumour vascularity has been shown to be a strong prognostic factor and to correlate with aggressiveness and metastatic potential in many cancers. High MVD has been associated with a poorer outcome in some studies of CRC (Saclarides *et al*, 1994; Tanigawa *et al*, 1997). Our results concur with this, with high MVD primary cancers and liver metastases having a reduced disease-free survival. A trend to decreased overall survival was also seen, but this did not reach statistical significance, and may reflect small patient numbers and non-cancer related deaths in a typically elderly CRC cohort.

Several methods have been described for determining tumour angiogenesis, including the use of Chalkley count (Hlatky *et al*, 2002), endothelial cell proliferation fraction (Hlatky *et al*, 2002), and pericyte coverage index (Yonenaga *et al*, 2005). The use of CD31 MVD analysis as an indicator of angiogenesis is well described. In this study, MVD analysis was performed using digital image analysis in preference to traditional tumour hotspot MVD measurement, which has been shown to be unreliable in assessing net angiogenic activity (Folkman, 1990; Takahashi *et al*, 1997; Hlatky *et al*, 2002; Subarsky and Hill, 2003). Our results proved to be reliable and reproducible on statistical testing.

One anomaly that emerges from our study is the unexpected finding of a higher MVD in pushing primary cancers as compared to their infiltrative counterparts. As pushing cancers are generally regarded as having a better prognosis, which we confirmed for disease-free survival, one might expect them to be associated with a lower MVD. Studies have also shown the pushing margin in primary cancers to have a better prognosis than the infiltrative margin tumours (Jass *et al*, 1987). This proved not to be the case and was probably a reflection of the cohort studied which was of advanced CRC with liver metastases, therefore is not representative of general spectrum CRC. One explanation could be the loss of significance between pushing and infiltrative margins with advanced CRC. Alternatively infiltrative primary and noncapsulated liver metastases may be less likely amenable to resection. The influence of the invasive pattern of the primary tumour and liver metastases on the resectability of the liver metastases has yet to be looked at.

This cohort was deliberately selected to comprise a high proportion of advanced cancers with liver metastasis, with >50% of patients having synchronous liver disease. It has been previously reported that patients who present with metastases at the time of surgery for primary colon cancer have higher vessel counts than patients with no evidence of metastases (Takahashi *et al*, 1997). Thus the high MVD observed in our pushing cohort may be a reflection of advanced disease stage.

Although the existence of two invasive phenotypes in CRC is now well- recognised and their prognostic implications proven, the biological explanation for their existence remains unexplained. The finding that pushing cancers tend to form capsulated liver metastases and infiltrative cancers tend to form non-capsulated liver metastases would argue for a genetic basis for these differences. However, one cannot ignore the influence of the tumour–stromal interaction on the invasive process, and this is highlighted by our finding of a significant difference in tumour margin MVD between pushing and infiltrative cancers. Such a difference was not apparent in liver metastases that reside in the highly vascularised liver parenchyma. One might hypothesise that the higher margin MVD observed in pushing cancers was indicative of better tumour oxygenation, with the assumption that microvessels are functionally competent (Subarsky and Hill, 2003). The corollary is that infiltrative cancers, with low margin MVD, are relatively hypoxic. If such a hypothesis is true, it might be expected that infiltrative cancers would express higher levels of hypoxia inducible factor (HIF-1*α*), which in turn would upregulate a number of downstream mediators including various proteases, such as MMP2 (Krishnamachary *et al*, 2003). These in turn might facilitate tumour invasion and account for their histological appearance. The upregulation of HIF-1*α* in CRC and liver metastases has been described previously (Mizukami *et al*, 2004), but its association with invasive phenotype requires further investigation.

The current study has highlighted the importance of the invasive margin in CRC progression and the potential value of tumour–stromal interactions, as assessed by MVD assessment, in prognostication. Further investigation is required to determine the biological mediators, which underlie CRC invasion, to enhance our understanding of the metastatic process, and to reveal potential new targets for therapeutic intervention.

## Figures and Tables

**Table 1:** Histological and clinical demographics of patients with CRC included in the study

**Clinicopathological details of patients (*n*=89)**	**Frequency**
Patient age mean	63
Range	(41–80)
	
*Patient sex*	
Male	26
Female	29
	
*Dukes stage at first presentation*	
A	1
B	18
C	36
	
*T stage*	
1	1
2	3
3	44
4	7
	
*N stage*	
0	27
1	19
2	9
	
*M stage*	
0	26
1	29
	
*Tumour site*	
Right-sided tumour	19
Left-sided tumour	13
Rectal tumour	23

CRC=colorectal cancer.

Variables relate to the time of surgical resection for primary CRC.

**Table 2:** Association between the invasive growth patterns of primary CRC(pushing/infiltrative) with MVD

	**Pushing MVD (95% CI)**	**Infiltrative MVD (95% CI)**	**Mann–Whitney *U*-test**
Primary tumour margin	82.4 (58.6–106.2)	51.3 (38.9–63.7)	*P*=0.034
Primary tumour center	62.5 (46.4–78.5)	43.4 (30.1–56.6)	*P*=0.077
Primary stromal margin	101.5 (74.4–128.6)	81.5 (59.5–103.6)	*P*=0.241

CRC=colorectal cancer; MVD=microvessel density.
